# Supplementary material for: Deletion of wfs1 Impairs Oligodendrocyte Precursor Cells Dorsal Distribution and Myelination Through the wfs1-hmgcs1 Axis in Zebrafish
Source: Biology (Basel). 2026 Aug 16;15(16):1402. doi: 10.3390/biology15161402 (PMC13510151; doi:10.3390/biology15161402)
Supplement: Supplementary file 1 [file biology-15-01402-s001.zip › File S1-Transcriptome sequencing data and differentially expressed genes (DEGs) in wfs1-- zebrafish at 5 dpf/1.GO/wfs1KOvsWT/GSEA/WT/EXORIBONUCLEASE_ACTIVITY/EXORIBONUCLEASE_ACTIVITY(GO_0004532).html]

Details for gene set EXORIBONUCLEASE\_ACTIVITY(GO:0004532)[GSEA]


|  || Dataset | deg.sample |
| Phenotype | sample.cls |
| Upregulated in class | WT |
| GeneSet | EXORIBONUCLEASE\_ACTIVITY(GO:0004532) |
| Enrichment Score (ES) | -0.59237933 |
| Normalized Enrichment Score (NES) | -1.7049706 |
| Nominal p-value | 0.0 |
| FDR q-value | 0.34162566 |
| FWER p-Value | 0.89 |
Table: GSEA Results Summary

  

Fig 1: Enrichment plot: EXORIBONUCLEASE\_ACTIVITY(GO:0004532)
  
Profile of the Running ES Score & Positions of GeneSet Members on the Rank Ordered List

  

| PROBE | DESCRIPTION   (from dataset) | GENE SYMBOL | GENE\_TITLE | RANK IN GENE LIST | RANK METRIC SCORE | RUNNING ES | CORE ENRICHMENT || 1 | 572044 | nocta |  |  | 4220 | 0.790 | -0.0750 | No |
| 2 | 553641 | eri1 |  |  | 7292 | 0.467 | -0.1374 | No |
| 3 | 791461 | parn |  |  | 11145 | 0.237 | -0.2411 | No |
| 4 | 394008 | xrn1 |  |  | 11487 | 0.217 | -0.2360 | No |
| 5 | 566106 | dis3l2 |  |  | 12971 | 0.142 | -0.2722 | No |
| 6 | 792831 | pan2 |  |  | 14896 | 0.051 | -0.3289 | No |
| 7 | 393717 | smfn |  |  | 15572 | 0.019 | -0.3487 | No |
| 8 | 321940 | xrn2 |  |  | 15911 | 0.002 | -0.3592 | No |
| 9 | 568260 | toe1 |  |  | 16214 | -0.008 | -0.3680 | No |
| 10 | 560386 | cnot6b |  |  | 16755 | -0.024 | -0.3832 | No |
| 11 | 100003553 | dis3 |  |  | 18790 | -0.113 | -0.4389 | No |
| 12 | 406788 | cnot8 |  |  | 18995 | -0.121 | -0.4365 | No |
| 13 | 445066 | usb1 |  |  | 20710 | -0.199 | -0.4758 | No |
| 14 | 562704 | angel2 |  |  | 21293 | -0.230 | -0.4773 | No |
| 15 | 553667 | eri2 |  |  | 21471 | -0.239 | -0.4655 | No |
| 16 | 558447 | isg20l2 |  |  | 24703 | -0.430 | -0.5356 | No |
| 17 | 768119 | cnot7 |  |  | 26063 | -0.535 | -0.5394 | Yes |
| 18 | 560362 | dis3l |  |  | 27067 | -0.636 | -0.5246 | Yes |
| 19 | 692319 | pde12 |  |  | 29228 | -0.981 | -0.5210 | Yes |
| 20 | 569547 | angel1 |  |  | 29593 | -1.092 | -0.4531 | Yes |
| 21 | 394064 | exosc10 |  |  | 30406 | -1.451 | -0.3732 | Yes |
| 22 | 553665 | cnot6l |  |  | 31268 | -2.362 | -0.2286 | Yes |
| 23 | 100534720 | isg20 |  |  | 31641 | -3.413 | 0.0078 | Yes |
Table: GSEA details
[plain text format]

  

Fig 2: EXORIBONUCLEASE\_ACTIVITY(GO:0004532)
  
Blue-Pink O' Gram in the Space of the Analyzed GeneSet

  

Fig 3: EXORIBONUCLEASE\_ACTIVITY(GO:0004532): Random ES distribution
  
Gene set null distribution of ES for
 **EXORIBONUCLEASE\_ACTIVITY(GO:0004532)**
